# Supplementary material for: Diagnostic and Prognostic Potential of SH3YL1 and NOX4 in Muscle-Invasive Bladder Cancer
Source: Int J Mol Sci. 2025 Apr 22;26(9):3959. doi: 10.3390/ijms26093959 (PMC12071612; doi:10.3390/ijms26093959)
Supplement: Supplementary file 1 [file ijms-26-03959-s001.zip › Figure S1.pdf]

Supplementary Figure S1. Expression levels of *SH3YL1* and *NOX4* across bladder cancer subtypes.

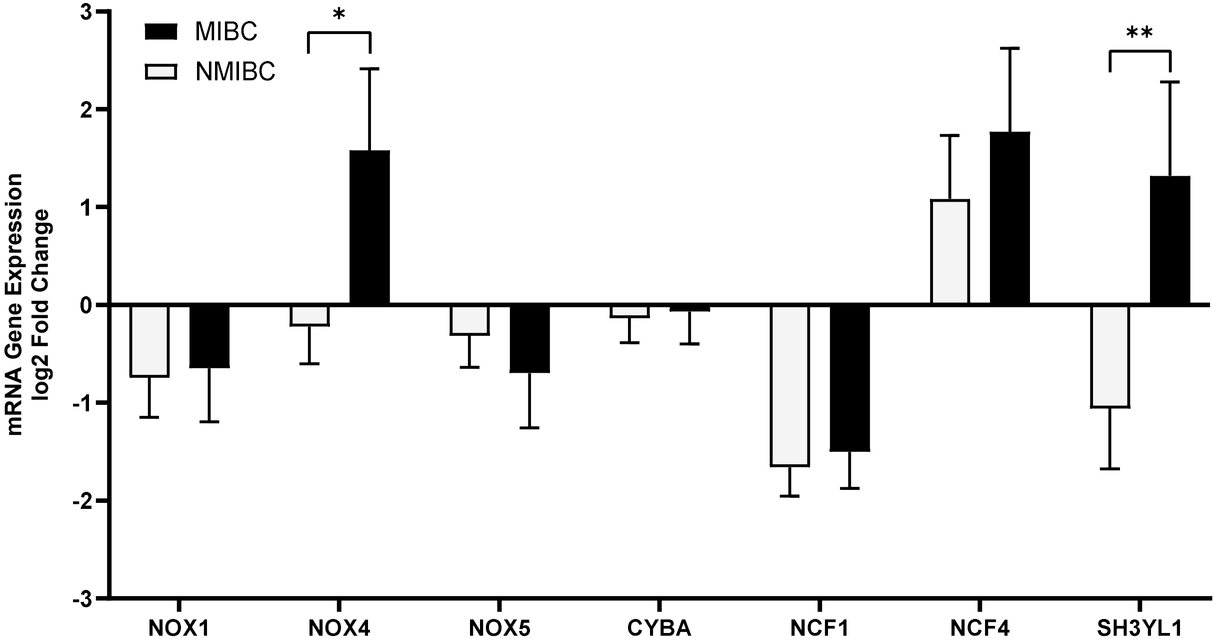

Figure S1. *SH3YL1* and *NOX4* mRNA expression levels were compared between non-muscle-invasive bladder cancer (NMIBC), and muscle-invasive bladder cancer (MIBC). Data are presented as the mean ± standard deviation (SD). ***p* < 0.05 and **p < 0.01 indicate statistically significant differences between groups.
