# Supplementary material for: Diagnostic and Prognostic Potential of SH3YL1 and NOX4 in Muscle-Invasive Bladder Cancer
Source: Int J Mol Sci. 2025 Apr 22;26(9):3959. doi: 10.3390/ijms26093959 (PMC12071612; doi:10.3390/ijms26093959)
Supplement: Supplementary file 1 [file ijms-26-03959-s001.zip › Figure S2.pdf]

Supplementary Figure S2. Kaplan-Meier survival analysis of *SH3YL1* expression in NMIBC.

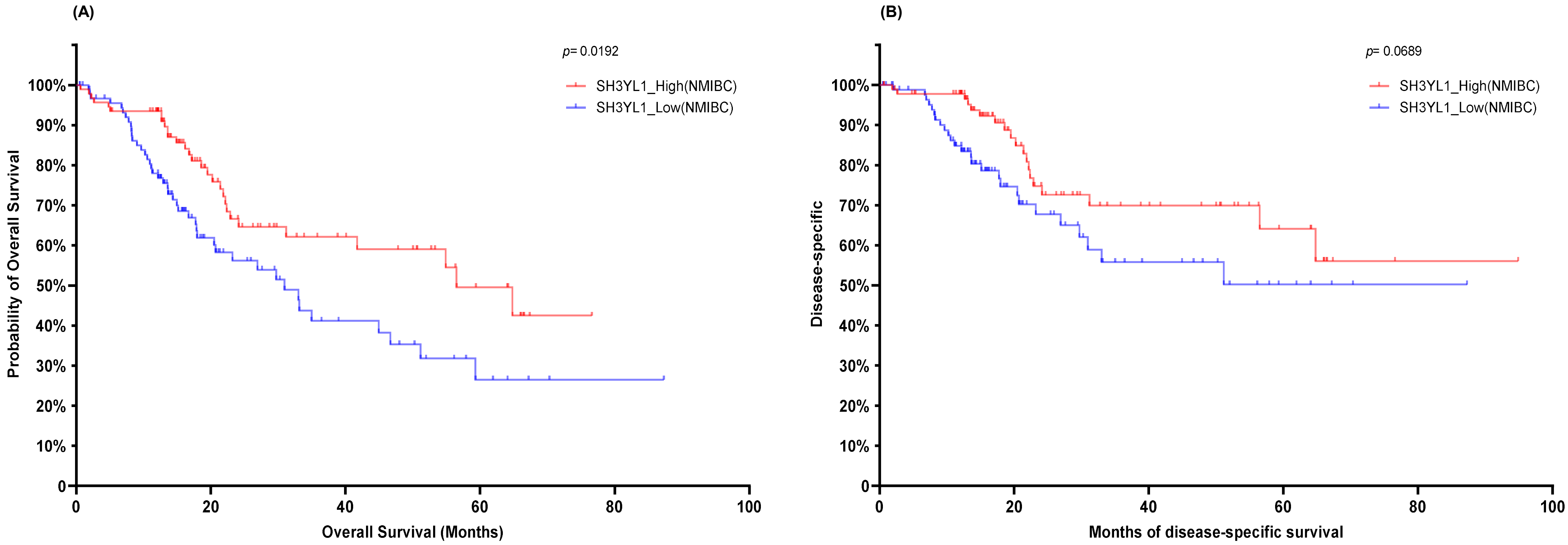

Figure S2. Kaplan-Meier survival analysis of *SH3YL1* expression in NMIBC. (A) Overall survival (OS) and (B) disease-specific survival (DSS) curves for NMIBC patients. While low *SH3YL1* expression was marginally associated with worse OS (p = 0.0192), the association with DSS was not statistically significant (p = 0.0689), suggesting limited prognostic value in early-stage disease.
