# Supplementary material for: Diagnostic and Prognostic Potential of SH3YL1 and NOX4 in Muscle-Invasive Bladder Cancer
Source: Int J Mol Sci. 2025 Apr 22;26(9):3959. doi: 10.3390/ijms26093959 (PMC12071612; doi:10.3390/ijms26093959)
Supplement: Supplementary file 1 [file ijms-26-03959-s001.zip › Figure S3.pdf]

Supplementary Figure S3. Kaplan-Meier Survival Analysis of *NOX4* Expression Within NMIBC and MIBC Groups

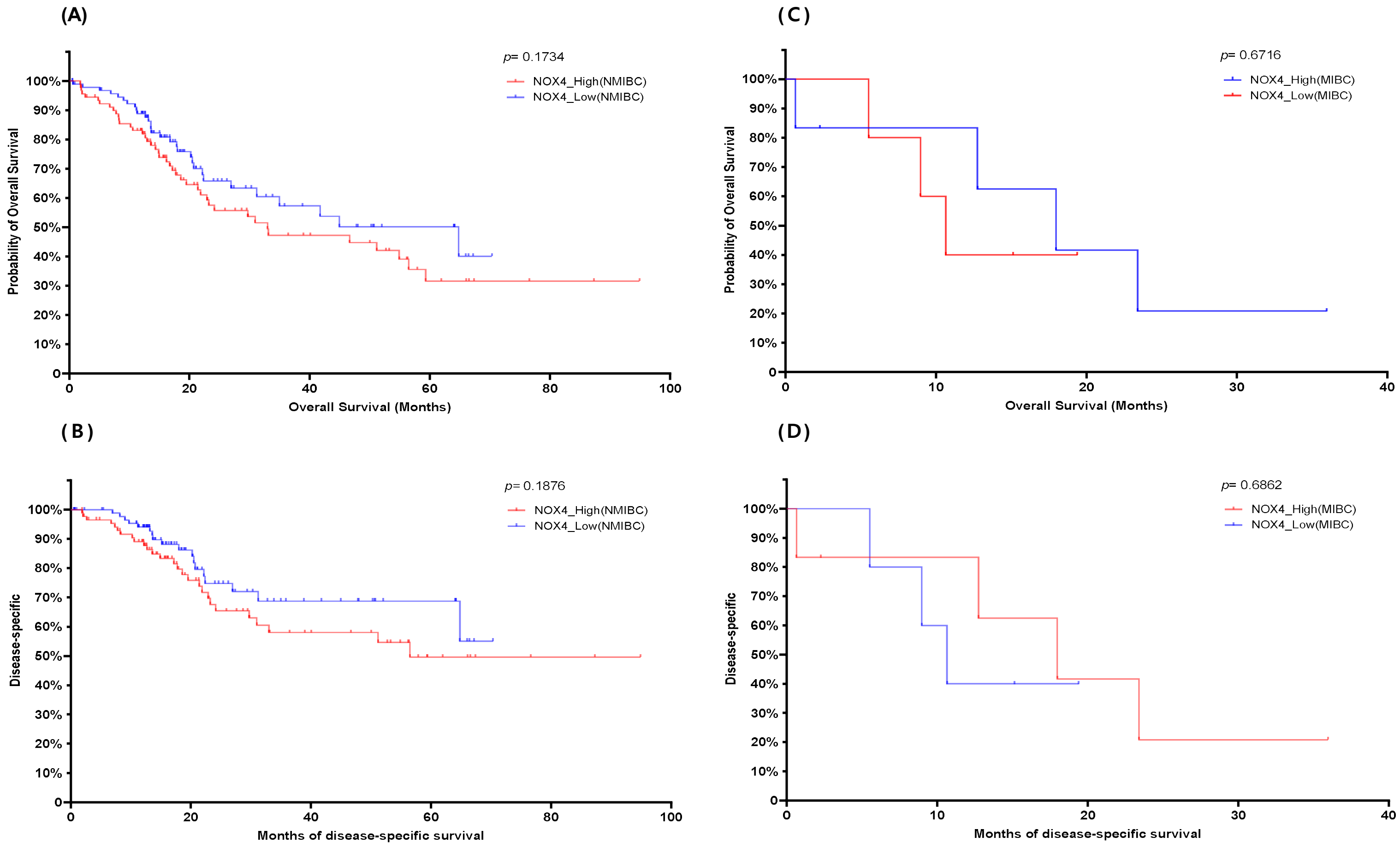

Figure S3. Kaplan-Meier survival analysis for *NOX4* expression levels within NMIBC and MIBC patient subgroups. (A) Overall survival (OS) for *NOX4*_High versus *NOX4*_Low in NMIBC patients. (B) Disease-specific survival (DSS) for *NOX4*_High versus *NOX4*_Low in NMIBC patients. (C) Overall survival (OS) for *NOX4*_High versus *NOX4*_Low in MIBC patients. (D) Disease-specific survival (DSS) for *NOX4*_High versus *NOX4*_Low in MIBC patients. The p-values were calculated using the log-rank test, indicating statistical significance where applicable.
