# Supplementary material for: Diagnostic and Prognostic Potential of SH3YL1 and NOX4 in Muscle-Invasive Bladder Cancer
Source: Int J Mol Sci. 2025 Apr 22;26(9):3959. doi: 10.3390/ijms26093959 (PMC12071612; doi:10.3390/ijms26093959)
Supplement: Supplementary file 1 [file ijms-26-03959-s001.zip › Figure S4.pdf]

Supplementary Figure S4. Kaplan-Meier Survival Analysis Comparing *NOX4* Expression Between NMIBC and MIBC Groups

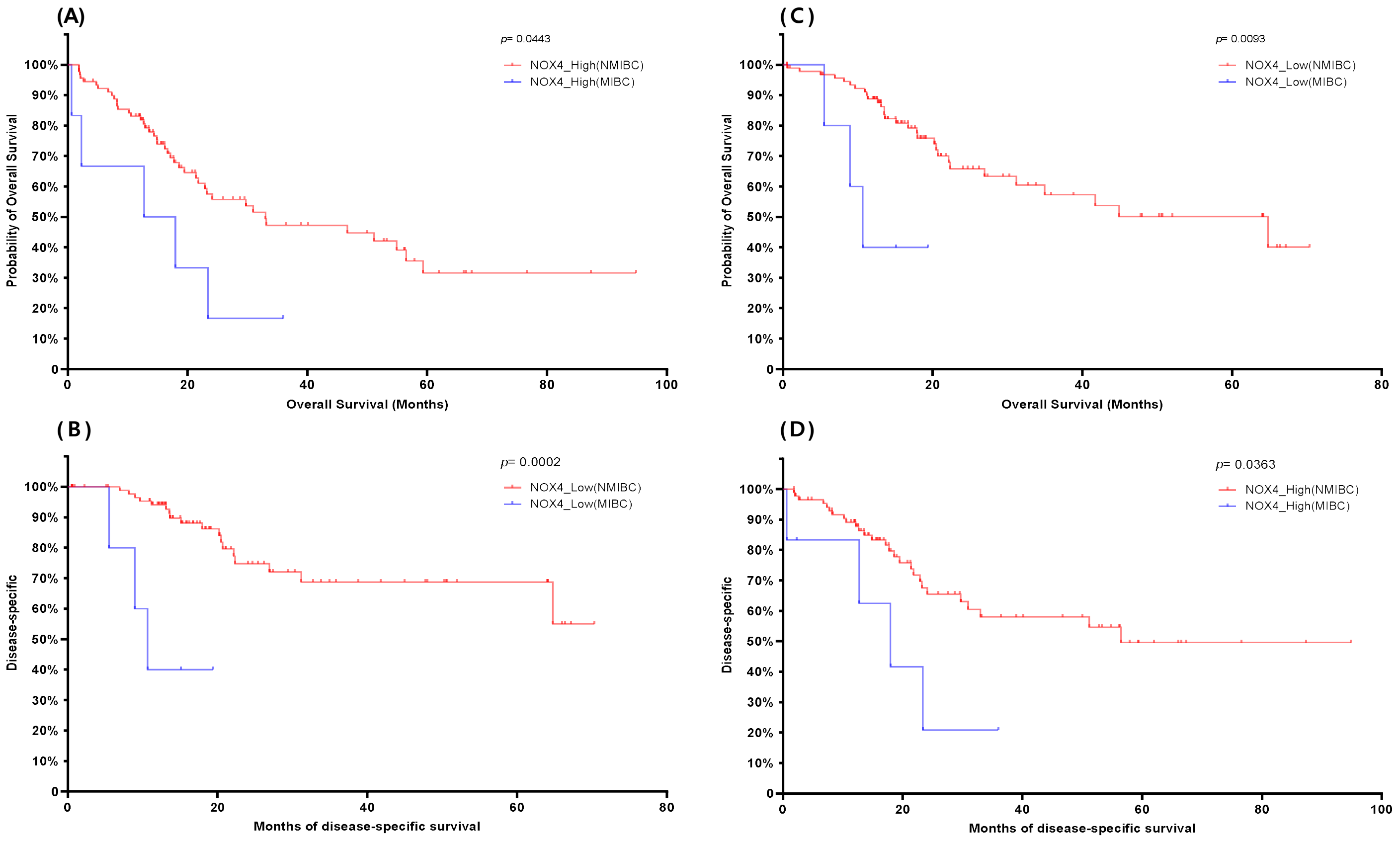

Figure S4. Kaplan-Meier survival analysis comparing *NOX4* expression between NMIBC and MIBC patient subgroups. (A) Overall survival (OS) between *NOX4*_High in NMIBC and MIBC. (B) Disease-specific survival (DSS) between *NOX4*_High in NMIBC and MIBC. (C) Overall survival (OS) between *NOX4*_Low in NMIBC and MIBC. (D) Disease-specific survival (DSS) between *NOX4*_Low in NMIBC and MIBC. The p-values were calculated using the log-rank test to assess statistical significance. This analysis evaluates the differential impact of *NOX4* expression on survival outcomes across bladder cancer subtypes.
