# Supplementary material for: Diagnostic and Prognostic Potential of SH3YL1 and NOX4 in Muscle-Invasive Bladder Cancer
Source: Int J Mol Sci. 2025 Apr 22;26(9):3959. doi: 10.3390/ijms26093959 (PMC12071612; doi:10.3390/ijms26093959)
Supplement: Supplementary file 1 [file ijms-26-03959-s001.zip › Table S1.pdf]

Table S1. Clinical Characteristics of MIBC, NMIBC, and Normal Samples

	**MIBC (n=16)**	**NMIBC (n=44)**	**NORMAL (n=20)**
**Age**			
Median	62.1	64.8	61.2
Range	28.6 - 82.4	40.0 - 87.1	32.4 - 81.2
**Gender**			
Male	14 (87.5%)	36 (81.8%)	16 (80%)
Female	2 (12.5%)	8 (18.2%)	4 (20%)
**Stage**			
Ta	1 (6.8%)	23 (52.3%)	
I	2 (12.5%)	20 (45.4%)	
II	9 (56.2%)	1 (2.3%)	
III	2 (12.5%)	0 (0%)	
IV	2 (12.5%)	0 (0%)	
**Grade**			
I	2 (12.5%)	5 (11.4%)	
II	1 (6.8%)	24 (54.5%)	
III	13 (81.2%)	15 (34.1%)	
IV	0 (0%)	0 (0%)	
