# Supplementary material for: Diagnostic and Prognostic Potential of SH3YL1 and NOX4 in Muscle-Invasive Bladder Cancer
Source: Int J Mol Sci. 2025 Apr 22;26(9):3959. doi: 10.3390/ijms26093959 (PMC12071612; doi:10.3390/ijms26093959)
Supplement: Supplementary file 1 [file ijms-26-03959-s001.zip › Table S2.pdf]

Table S2. Metascape Node Details for Figure 2B

Canonical Name	Category	Category ID	Description	Enrichment	FirstInGroup ByEnrichment	FirstInGroup ByLogP	GROUP _ID	Hits	id	Log(q-value)	LogP	name
GO:0042554	GO Biological Processes	19	superoxide anion generation	456.45949	1	1	1	CYBA|CYBB|NCF2|NCF4|SH3PXD2A|NOXA1|NOX1|DUOX2|NOX4|NOX3|DUOX1|NOX5|NOXO1	67	-28.3692	-32.7126	67
GO:0006897	GO Biological Processes	19	endocytosis	13.40269	1	1	7	NCF2|NCF4|TRIP10|FCHSD2|FNBP1|PACSIN1|FNBP1L|NOSTRIN	66	-4.1116	-6.9636	66
GO:0044248	GO Biological Processes	19	cellular catabolic process	5.33351	1	1	9	PIK3C2B|DUOX2|DUOX1|FNBP1L|VPS37B	65	-0.2666	-2.6415	65
hsa05132	KEGG Pathway	24	Salmonella infection	13.42939	1	1	8	PIK3C2B|PIK3C2G|WASL|ABI1	64	-1.1678	-3.6599	64
WP408	WikiPathways	27	Oxidative stress response	127.68098	1	1	3	CYBB|NOX1|NOX4|NOX3|NOX5	63	-6.3246	-9.3892	63
R-HSA-199991	Reactome Gene Sets	6	Membrane Trafficking	11.94370	1	1	6	WASL|TRIP10|FNBP1|PACSIN1|UBAP1|FNBP1L|VPS37B|MVB12B|SH3D19	62	-4.4706	-7.3826	62
R-HSA-9658195	Reactome Gene Sets	6	Leishmania infection	41.10705	1	1	2	CYBA|WASL|ABI1|NOXA1|NOX1|NOXO1|WIPF2|WIPF3	61	-7.6195	-10.7868	61
R-HSA-9664420	Reactome Gene Sets	6	Killing mechanisms	280.89815	1	1	5	CYBA|NOXA1|NOX1|NOXO1	60	-6.1041	-9.0858	60
R-HSA-9013148	Reactome Gene Sets	6	CDC42 GTPase cycle	38.05717	1	1	4	WASL|SH3PXD2A|FNBP1|DNMBP|FNBP1L|WIPF2|WIPF3	59	-6.1908	-9.2332	59
GO:0061919	GO Biological Processes	19	process utilizing autophagic mechanism	7.73114	0	0	9	PIK3C2B|FNBP1L|VPS37B	58	0.0000	-2.1668	58
GO:0006914	GO Biological Processes	19	autophagy	7.73114	0	0	9	PIK3C2B|FNBP1L|VPS37B	57	0.0000	-2.1668	57
GO:0006644	GO Biological Processes	19	phospholipid metabolic process	6.94528	0	0	8	PIK3C2B|PIK3C2G|SH3YL1	56	0.0000	-2.0397	56
GO:0046486	GO Biological Processes	19	glycerolipid metabolic process	7.06169	0	0	8	PIK3C2B|PIK3C2G|SH3YL1	55	0.0000	-2.0594	55
GO:0006650	GO Biological Processes	19	glycerophospholipid metabolic process	8.62827	0	0	8	PIK3C2B|PIK3C2G|SH3YL1	54	0.0000	-2.2984	54
GO:0090407	GO Biological Processes	19	organophosphate biosynthetic process	6.18491	0	0	8	PIK3C2B|PIK3C2G|SH3YL1|RFK	53	-0.0757	-2.4147	53
GO:0008654	GO Biological Processes	19	phospholipid biosynthetic process	10.44663	0	0	8	PIK3C2B|PIK3C2G|SH3YL1	52	-0.1740	-2.5306	52
GO:0045017	GO Biological Processes	19	glycerolipid biosynthetic process	11.28609	0	0	8	PIK3C2B|PIK3C2G|SH3YL1	51	-0.2551	-2.6254	51
GO:0046474	GO Biological Processes	19	glycerophospholipid biosynthetic process	12.51526	0	0	8	PIK3C2B|PIK3C2G|SH3YL1	50	-0.3638	-2.7529	50
GO:0046488	GO Biological Processes	19	phosphatidylinositol metabolic process	16.00053	0	0	8	PIK3C2B|PIK3C2G|SH3YL1	49	-0.6555	-3.0593	49
GO:0006661	GO Biological Processes	19	phosphatidylinositol biosynthetic process	20.06415	0	0	8	PIK3C2B|PIK3C2G|SH3YL1	48	-0.8881	-3.3450	48
GO:0098657	GO Biological Processes	19	import into cell	9.77037	0	0	7	NCF2|NCF4|TRIP10|FCHSD2|FNBP1|PACSIN1|FNBP1L|NOSTRIN	47	-3.2549	-5.9261	47
GO:0061024	GO Biological Processes	19	membrane organization	7.84423	0	0	6	WASL|FCHSD2|PACSIN1|UBAP1|FNBP1L|BAIAP2L1|VPS37B	46	-2.0520	-4.6029	46
GO:0043162	GO Biological Processes	19	ubiquitin-dependent protein catabolic process via the multivesicular body sorting pathway	68.32658	0	0	6	UBAP1|VPS37B|MVB12B	45	-2.3700	-4.9357	45
R-HSA-9615710	Reactome Gene Sets	6	Late endosomal microautophagy	72.23095	0	0	6	UBAP1|VPS37B|MVB12B	44	-2.4220	-5.0095	44
R-HSA-917729	Reactome Gene Sets	6	Endosomal Sorting Complex Required For Transport (ESCRT)	79.00260	0	0	6	UBAP1|VPS37B|MVB12B	43	-2.5337	-5.1288	43
R-HSA-162588	Reactome Gene Sets	6	Budding and maturation of HIV virion	87.17529	0	0	6	UBAP1|VPS37B|MVB12B	42	-2.6485	-5.2605	42
R-HSA-175474	Reactome Gene Sets	6	Assembly Of The HIV Virion	158.00521	0	0	6	UBAP1|VPS37B|MVB12B	41	-3.3811	-6.0704	41
R-HSA-174495	Reactome Gene Sets	6	Synthesis And Processing Of GAG, GAGPOL Polyproteins	180.57738	0	0	6	UBAP1|VPS37B|MVB12B	40	-3.5045	-6.2568	40
R-HSA-174490	Reactome Gene Sets	6	Membrane binding and targetting of GAG proteins	180.57738	0	0	6	UBAP1|VPS37B|MVB12B	39	-3.5045	-6.2568	39
R-HSA-5653656	Reactome Gene Sets	6	Vesicle-mediated transport	11.25260	0	0	6	WASL|TRIP10|FNBP1|PACSIN1|UBAP1|FNBP1L|VPS37B|MVB12B|SH3D19	38	-4.2658	-7.1620	38
GO:2000377	GO Biological Processes	19	regulation of reactive oxygen species metabolic process	17.08164	0	0	5	CYBA|NOXA1|NOXO1	37	-0.7145	-3.1415	37
WP231	WikiPathways	27	TNF alpha signaling	37.87391	0	0	5	CYBA|NOX1|RFK|NOXO1	36	-2.7851	-5.4209	36
R-HSA-9673324	Reactome Gene Sets	6	WNT5:FZD7-mediated leishmania damping	280.89815	0	0	5	CYBA|NOXA1|NOX1|NOXO1	35	-6.1041	-9.0858	35

GO:0120035	GO Biological Processes	19	regulation of plasma membrane bounded cell projection organization	5.22601	0	0	4	WASL|SH3YL1|PACSIN1|FNBP1L	34	0.0000	-2.1578	34
hsa05131	KEGG Pathway	24	Shigellosis	10.15295	0	0	4	WASL|FNBP1|FNBP1L	33	-0.1436	-2.4957	33
GO:0060491	GO Biological Processes	19	regulation of cell projection assembly	12.21296	0	0	4	WASL|SH3YL1|FNBP1L	32	-0.3431	-2.7227	32
GO:0120032	GO Biological Processes	19	regulation of plasma membrane bounded cell projection assembly	12.33211	0	0	4	WASL|SH3YL1|FNBP1L	31	-0.3504	-2.7347	31
GO:0007009	GO Biological Processes	19	plasma membrane organization	16.74227	0	0	4	PACSIN1|FNBP1L|BAIAP2L1	30	-0.7023	-3.1163	30
GO:0044089	GO Biological Processes	19	positive regulation of cellular component biogenesis	8.18150	0	0	4	WASL|FCHSD2|POLDIP2|FNBP1L|BAIAP2L1	29	-0.9975	-3.4717	29
R-HSA-9013406	Reactome Gene Sets	6	RHOQ GTPase cycle	42.84887	0	0	4	WASL|TRIP10|FNBP1	28	-1.7847	-4.3219	28
R-HSA-8856828	Reactome Gene Sets	6	Clathrin-mediated endocytosis	28.85940	0	0	4	WASL|TRIP10|FNBP1|PACSIN1|FNBP1L	27	-3.3840	-6.0939	27
R-HSA-9013409	Reactome Gene Sets	6	RHOJ GTPase cycle	61.28687	0	0	4	WASL|FNBP1|FNBP1L|WIPF2	26	-3.5045	-6.2633	26
GO:0006954	GO Biological Processes	19	inflammatory response	5.82172	0	0	3	CYBA|CYBB|NOX1|NOX4	25	0.0000	-2.3217	25
WP2059	WikiPathways	27	Alzheimer 39 s disease and miRNA effects	9.43315	0	0	3	CYBB|NOX1|NOX4	24	-0.0715	-2.4062	24
WP5124	WikiPathways	27	Alzheimer 39 s disease	9.61248	0	0	3	CYBB|NOX1|NOX4	23	-0.0857	-2.4291	23
GO:0001819	GO Biological Processes	19	positive regulation of cytokine production	6.67481	0	0	3	CYBA|CYBB|NOX1|NOX5	22	-0.1740	-2.5329	22
hsa04933	KEGG Pathway	24	AGE-RAGE signaling pathway in diabetic complications	25.03053	0	0	3	CYBB|NOX1|NOX4	21	-1.1409	-3.6269	21
WP5420	WikiPathways	27	ADHD and autism ASD pathways	11.35707	0	0	3	NOX1|NOX4|NOX3|RFK|NOX5	20	-1.6165	-4.1338	20
WP4313	WikiPathways	27	Ferroptosis	38.30429	0	0	3	CYBB|NOX1|NOX4	19	-1.6520	-4.1759	19
R-HSA-2029480	Reactome Gene Sets	6	Fcgamma receptor (FCGR) dependent phagocytosis	39.19509	0	0	2	WASL|ABI1|WIPF2|WIPF3	18	-2.8295	-5.4804	18
GO:0007015	GO Biological Processes	19	actin filament organization	18.18765	0	0	2	WASL|ABI1|PACSIN1|BAIAP2L1|NOSTRIN|WIPF3	17	-3.3811	-6.0617	17
R-HSA-2029482	Reactome Gene Sets	6	Regulation of actin dynamics for phagocytic cup formation	55.25865	0	0	2	WASL|ABI1|WIPF2|WIPF3	16	-3.3811	-6.0808	16
R-HSA-9664422	Reactome Gene Sets	6	FCGR3A-mediated phagocytosis	57.13183	0	0	2	WASL|ABI1|WIPF2|WIPF3	15	-3.4194	-6.1395	15
R-HSA-9664417	Reactome Gene Sets	6	Leishmania phagocytosis	57.13183	0	0	2	WASL|ABI1|WIPF2|WIPF3	14	-3.4194	-6.1395	14
R-HSA-9664407	Reactome Gene Sets	6	Parasite infection	57.13183	0	0	2	WASL|ABI1|WIPF2|WIPF3	13	-3.4194	-6.1395	13
GO:0030029	GO Biological Processes	19	actin filament-based process	10.85597	0	0	2	WASL|TRIP10|ABI1|PACSIN1|FNBP1L|BAIAP2L1|NOSTRIN|WIPF3	12	-3.5045	-6.2693	12
R-HSA-5663213	Reactome Gene Sets	6	RHO GTPases Activate WASPs and WAVEs	93.63272	0	0	2	WASL|ABI1|WIPF2|WIPF3	11	-4.1528	-7.0190	11
R-HSA-9824443	Reactome Gene Sets	6	Parasitic Infection Pathways	41.10705	0	0	2	CYBA|WASL|ABI1|NOXA1|NOX1|NOXO1|WIPF2|WIPF3	10	-7.6195	-10.7868	10
R-HSA-195258	Reactome Gene Sets	6	RHO GTPase Effectors	31.01943	0	0	1	CYBA|CYBB|NCF2|NCF4|WASL|ABI1|NOXA1|NOX1|NOX3|NOXO1|WIPF2|WIPF3	9	-11.3723	-14.7157	9
R-HSA-9013423	Reactome Gene Sets	6	RAC3 GTPase cycle	89.64835	0	0	1	CYBA|CYBB|NCF2|NCF4|ABI1|NOXA1|NOX1|NOX3|BAIAP2L1|NOXO1	8	-13.5373	-16.9264	8
R-HSA-5668599	Reactome Gene Sets	6	RHO GTPases Activate NADPH Oxidases	280.89815	0	0	1	CYBA|CYBB|NCF2|NCF4|NOXA1|NOX1|NOX3|NOXO1	7	-14.4679	-17.9081	7
R-HSA-9716542	Reactome Gene Sets	6	Signaling by Rho GTPases, Miro GTPases and RHOBTB3	21.00900	0	0	1	CYBA|CYBB|NCF2|NCF4|WASL|TRIP10|SH3PXD2A|ABI1|NOXA1|FNBP1|DNMBP|NOX1|NOX3|FNBP1L|BAIAP2L1|NOXO1|WIPF2|WIPF3	6	-16.0302	-19.5284	6
R-HSA-9013149	Reactome Gene Sets	6	RAC1 GTPase cycle	59.21637	0	0	1	CYBA|CYBB|NCF2|NCF4|WASL|ABI1|NOXA1|NOX1|NOX3|BAIAP2L1|NOXO1|WIPF2|WIPF3	5	-16.1025	-19.6677	5
R-HSA-194315	Reactome Gene Sets	6	Signaling by Rho GTPases	21.48513	0	0	1	CYBA|CYBB|NCF2|NCF4|WASL|TRIP10|SH3PXD2A|ABI1|NOXA1|FNBP1|DNMBP|NOX1|NOX3|FNBP1L|BAIAP2L1|NOXO1|WIPF2|WIPF3	4	-16.1025	-19.7017	4
R-HSA-9012999	Reactome Gene Sets	6	RHO GTPase cycle	33.78285	0	0	1	CYBA|CYBB|NCF2|NCF4|WASL|TRIP10|SH3PXD2A|ABI1|NOXA1|FNBP1|DNMBP|NOX1|NOX3|FNBP1L|BAIAP2L1|NOXO1|WIPF2|WIPF3	3	-19.4897	-23.2310	3
GO:0072593	GO Biological Processes	19	reactive oxygen species metabolic process	108.23598	0	0	1	CYBA|CYBB|NCF2|NCF4|SH3PXD2A|NOXA1|NOX1|DUOX2|NOX4|NOX3|DUOX1|RFK|NOX5|NOXO1	2	-21.1833	-25.0495	2
GO:0006801	GO Biological Processes	19	superoxide metabolic process	243.44506	0	0	1	CYBA|CYBB|NCF2|NCF4|SH3PXD2A|NOXA1|NOX1|DUOX2|NOX4|NOX3|DUOX1|NOX5|NOXO1	1	-24.2106	-28.2529	1
